# Development of a validated patient-reported symptom metric for pediatric Eosinophilic Esophagitis: qualitative methods

**DOI:** 10.1186/1471-230X-11-126

**Published:** 2011-11-18

**Authors:** James P Franciosi, Kevin A Hommel, Charles W DeBrosse, Allison B Greenberg, Alexandria J Greenler, J Pablo Abonia, Marc E Rothenberg, James W Varni

**Affiliations:** 1 Division of Gastroenterology, Hepatology and Nutrition, Cincinnati Children's Hospital Medical Center, Cincinnati, OH, USA; 2 Center for the Promotion of Treatment Adherence and Self-Management, Division of Behavioral Medicine and Clinical Psychology, Cincinnati Children's Hospital Medical Center, Cincinnati, OH, USA; 3 Division of Allergy and Immunology, Cincinnati Children's Hospital Medical Center, Cincinnati, OH, USA; 4 Clinical Trials Office, Cincinnati Children's Hospital Medical Center, Cincinnati, OH, USA; 5 Department of Pediatrics, College of Medicine, Department of Landscape Architecture and Urban Planning, College of Architecture, Texas A&M University, College Station, TX, USA

## Abstract

**Background:**

Previous attempts to measure symptoms in pediatric Eosinophilic Esophagitis (EoE) have not fully included patients and parents in the item development process. We sought to identify and validate key patient self-reported and parent proxy-reported outcomes (PROs) specific to EoE.

**Methods:**

We developed methodology for focus and cognitive interviews based on the Food and Drug Administration (FDA) guidelines for PROs, the validated generic PedsQL™ guidelines, and the consolidated criteria for reporting qualitative research (COREQ). Both child (ages 8-12 and 13-18) and parent-proxy (ages 2-4, 5-7, 8-12, and 13-18) interviews were conducted.

**Results:**

We conducted 75 interviews to construct the new instrument. Items were identified and developed from individual focus interviews, followed by cognitive interviews for face and content validation. Initial domains of symptom frequency and severity were developed, and open-ended questions were used to generate specific items during the focus interviews. Once developed, the instrument construct, instructions, timeframe, scoring, and specific items were systematically reviewed with a separate group of patients and their parents during the cognitive interviews.

**Conclusions:**

To capture the full impact of pediatric EoE, both histologic findings and PROs need to be included as equally important outcome measures. We have developed the face and content validated Pediatric Eosinophilic Esophagitis Symptom Score (PEESS™ v2.0). The PEESS™ v2.0 metric is now undergoing multisite national field testing as the next iterative instrument development phase.

## Background

In pediatric eosinophilic esophagitis (EoE) there can be a wide range of symptoms: from the easily recognizable presentations of food bolus impaction and dysphagia to the less obvious feeding disorders and abdominal pain [1]. Most previous efforts to develop a questionnaire to measure pediatric EoE symptoms have focused on correlations between non-validated symptom scores and histologic outcomes, specifically peak esophageal eosinophil counts [2,3]. To date, there have been few validated instruments that capture symptoms as patient and parent proxy-reported outcomes (PROs) developed through patient focus interviews and cognitive interviews as recommended by the FDA and specific to pediatric EoE [1,4].

Pentiuk et al. developed the non-validated Pediatric Eosinophilic Esophagitis Symptom Score (PEESS version 1.0) utilizing expert opinion and focusing on correlations between symptom scores and histologic outcomes. In their study, Pentiuk and colleagues demonstrated that subjects with untreated EoE had higher PEESS scores than treated subjects; however, symptom score and histology were only weakly correlated [5]. Aceves and colleagues have also recently developed a symptom score through the modification of a metric utilized for acid peptic disorders [3]. However, this non-validated tool was again developed through expert opinion alone and focused on correlations to histology. In Aceves's study, the total symptom score was higher among patients with EoE and gastroesophageal reflux disease (GERD) than control patients (P < 0.001). However, only symptoms of dysphagia and anorexia/early satiety were capable of significantly discriminating EoE from GERD (P < 0.01). Patients with chief complaints other than dysphagia and anorexia did not have histology findings that directly correlated with the results of the modified symptoms scale.

These prior efforts to develop a symptom score for EoE depending solely on expert opinion have not fully taken into account patient perceptions of symptom severity or response to treatment, which are increasingly important to improving health outcomes and quality of life [4]. Therefore, there is a significant need for severity indices to be developed as patient self-report and parent proxy-reported outcomes. Well designed and validated PROs have been increasingly recognized as key outcome measures for the treatment of chronic disease over the past decade. For example, the recent pediatric asthma randomized clinical trial for the medication ciclesonide utilized both patient symptoms and quality of life (QOL) metrics as key outcome measures and demonstrated improvements in QOL and symptoms scores in the treated groups relative to the placebo controls [6]. The National Institutes of Health identified PROs as key components in the clinical research "toolbox" and have launched the Patient Reported Outcomes Measurement Information System (PROMIS, http://www.nihpromis.org/default.aspx) in an attempt to provide healthcare workers and researchers with instruments to objectively measure the disease characteristics that patients and their families deem critically important for their day-to-day health [7-14]. It is clear that a pediatric EoE symptom score would provide a valuable new tool with which to better analyze the outcomes in pediatric EoE that are relevant to families.

As a first phase in the development process of a new PRO instrument, content validity must be established. The FDA guidelines on PRO development state that, "item generation should include input from the target patient population to establish the items that reflect the concept of interest and contribute to its evaluation." To capture outcomes from the patients' perspective, in 2009 the FDA recommended that it is important to establish content validity through patient focus groups/interviews and cognitive think aloud and cognitive debriefing protocols before evaluating other measurement properties. Basic PRO validation requires feedback from patients and their parents early in the instrument development process to determine if the instrument's item content captures the disease features that pediatric patients and parents believe are important for the instrument to measure. Content validity is supported using qualitative research methodologies to investigate whether the PRO instrument and its respective items measure the symptoms of interest from the patients' perspective. These qualitative methods are an essential part of the instrument development process from the beginning of the process, and subsequently testing other measurement properties will not replace or rectify problems with eliciting patient and parent-proxy focused outcomes from the initiation phase [7]. In 2008, Flood and colleagues described their use of cognitive interviews for a new pediatric EoE metric (Symptom Questionnaire for Eosinophilic Esophagitis) developed by experts in the EoE field for patients age 8-17 years of age and caregivers of EoE patients ages 2-7 years of age [15]. Given that the metric developed by Flood and colleagues was developed by expert opinion initially with patient input in a single cohort only, it may be limited in reflecting the patient experience.

As the primary objective of this study, we sought to identify key EoE patient self-reported and parent proxy-reported symptoms through focus interviews, to develop an EoE PRO symptom metric, and to review the patient derived metric though cognitive interviews in a separate cohort. Content validation of the Pediatric Eosinophilic Esophagitis Symptoms Severity (PEESS™ v2.0) PRO metric was supported by this iterative process.

## Methods

All study protocols were reviewed and approved by the Institutional Review board at Cincinnati Children's Hospital Medical Center (CCHMC). All research described was compliant with the CCHMC ethical guidelines for clinical research and the Helsinki Declaration (http://www.wma.net/e/policy/b3.htm). Informed consent was obtained from all parents and assent for children ages 8-18 years of age.

FDA guidelines on PRO development, the validated generic PedsQL™ guidelines, and the consolidated criteria for reporting qualitative research (COREQ) were used to develop the methodology for content validation [7,13,16].

### Research Team

The research team was composed of experts in the field of EoE including specialty physicians from the fields of Allergy and Gastroenterology with advanced training in clinical research methodologies and two Ph.D. psychologists with specific expertise in qualitative research and PRO methodology. The entire team developed interview guidelines, and specific interviewers were trained in qualitative methodology. The research team reviewed transcriptions of the interview audiotapes.

### Participants

Participants were identified from local and referral populations with a clinicopathologic diagnosis of EoE based on chart review and family interview. All participants had a clinical diagnosis of EoE, and their histology reports were reviewed to identify at least one endoscopy with >= 15 eos/hpf isolated to the esophagus. As it was critical to assess patients' and families' concerns that were specific to EoE, not those related to other co-morbidities, pediatric patient participants were restricted to those with a diagnosis of EoE and without other co-morbidities, including clinical diagnoses of: gastroesophageal reflux (GERD), inflammatory bowel disease, celiac disease, psychiatric disorder, and/or therapy with psychiatric/behavioral medication.

### Focus Interviews

The methodologies for focus interviews (also termed concept elicitation interviews) have been described previously [12,17]. Briefly, our research team developed a script of semi-structured open-ended questions (Table 1). The content of the PEESS v1.0 was used only as a general outline for open-ended patient and parent-proxy questions. Interviewers facilitated the participants' answers by focusing discussion on the topics under consideration while being nondirective and nonevaluative. Interviews lasted approximately one hour, with age stratification in alignment with the PedsQL™ guidelines (http://www.pedsql.org), considered a gold standard for pediatric PRO metrics [12].

**Table 1:** Focus interview open-ended questions

Participant	Questions
	What symptoms do you (does your child) have that you relate to EoE?
	Not eating?
	Pain in chest?
	Burning in chest?
**Child (Parent)**	Trouble swallowing (eating food)?
	Vomiting/throwing up?
	What is the most frequent symptom? How often does this occur?
	What is the worst symptom? How often does this occur?
	How often to do you call your (your child's) doctor?
	Because of your (your child's) symptoms, do you (s/he) have trouble in school? Work? Playing with friends?
	What trouble do you (your child) have eating food?

All sessions were audio-recorded, transcribed verbatim, and analyzed by the research team. Responses were grouped according to domains of interest, age ranges, and patient vs. parent proxy groups. Content and themes were then derived by consensus among the research team. Disagreements were minor and easily resolved by further discussion and then consensus was reached within the group.

### Expert Opinion

Items derived from the focus interviews were integrated into a preliminary draft of the PEESS™ version 2.0. Local (authors) and national (listed in acknowledgements) EoE experts in the fields of allergy, gastroenterology, and psychology then reviewed this draft. To retain the focus of the PEESS™ v2.0 on patient self-reported and parent proxy-reported outcomes, experts were allowed only to discuss general concerns and suggest (not delete) additional items. This additional information was then reviewed with a separate cohort of subjects in the cognitive interview phase. This step is a critical distinction from previous EoE metrics [15].

### Cognitive Interviews

**Figure 1:**
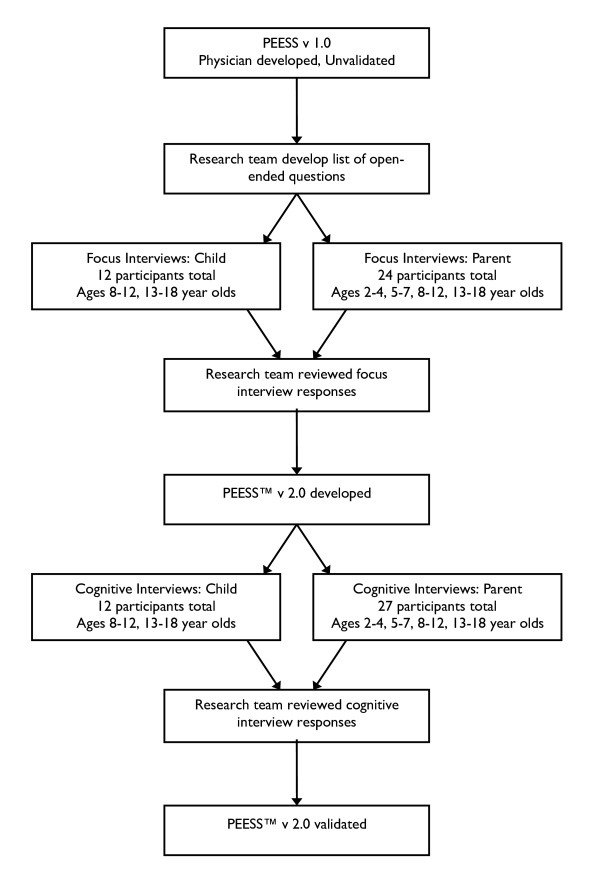
**Summary of content validation methodology**. Focus interview transcripts of pediatric patients with EoE and their parents were used to develop the items and domains for the PEESS™ v2.0. Cognitive interviewing was conducted with separate cohorts of pediatric patients and their parents in the 8-12 and 13-18 year old age groups, while parent proxy-reports were also obtained in the 2-4 year old and 5-7 year old age groups.

Cognitive interviews were designed to elicit information regarding the clarity and rationale of the directions, individual items, domains, and response choices, as well as overall comments on the relevance and complexity of the questionnaire. Participants completed and reviewed the PEESS™ version 2.0 preliminary draft derived from the focus interviews and provided feedback utilizing the previously described respondent debriefing methodology [13]. Briefly, cognitive probes and items generated by focus interview subjects and national experts were assimilated into a protocol from the existing methodological literature by Varni and colleagues (Table 2). An item-by-item summary of each section of the questionnaire, including recommendations for modifications, was prepared from transcribed audiotapes and interviewer notes. Using the same methodology as described for the focus interviews, items and content generated were revised. Reading level was assessed using the Flesch Reading Easiness and the Flesch-Kincaid Grade Level scores and used to further revise question language and grammar. A summary of the content validation methodology is provided in Figure 1.

**Table 2:** Cognitive interview respondent debriefing

Subject	Question
**Directions**	How would you make the directions more clear/easy to understand?
	What does "in the past month" mean to you?
	When you see "the past month", what days did you include?
**Items**	In your own words, what do you think this question is asking?
	What does this question mean to you? What did you think of when answering this question?
	Was this question easy to understand? Are there any specific words that are difficult to understand?
	How would you change the words to make it more clear?
	Was this item hard to answer? If yes, why?
	How did you choose your answer?
**Domains**	In your own words, what do you think this group of questions is asking about?
	How do you think these items are related?
	Are there any questions that do not belong in this group?
**Response** **Choices**	What do you think about the response choices?
	How would you make the response choices clearer or easier to understand?
**Overall** **Assessment**	Are there things that we forgot to ask about that you think are important?
	Overall thoughts/opinions of the questionnaire?
	Anything you would change in the questionnaire as a whole?

## Results

Basic participant demographics of the focus interview and cognitive interview cohorts are provided in Table 3.

**Table 3:** Demographics of focus interview (FI) and cognitive interview (CI) cohorts

	Ages 2-4	Ages 5-7	Ages 8-12	Ages 13-18
**Total sample (FI,CI)**	13 (6,7)	12 (7,5)	12 (6,6)	13 (7,6)
**Male % (FI, CI)**	61.5% (3,5)	83% (6,4)	50% (2,4)	61.5% (4,4)
**Caucasian % (FI, CI)**	77% (5,5)	83% (5,5)	83% (6,4)	92% (7,5)
**Non-Hispanic % (FI, CI)**	92% (5,7)	100% (7,5)	100% (6,6)	92% (6,6)
**Dietary food restrictions for EoE % (FI, CI)**	46% (2,4)	75% (5,4)	50% (1,5)	54% (1,6)
**Swallowed steroids for EoE % (FI, CI)**	46% (4,2)	42% (4,1)	58% (2,5)	38% (4,1)
**Food allergies % (FI, CI)**	69% (5,4)	75% (5,4)	58% (1,6)	31% (0,4)
**Atopic disease % (FI, CI)**	61.5% (3,5)	75% (5,4)	42% (2,3)	92% (6,6)

### Focus Interviews Patient Self-Report

A total of 36 participants were interviewed, divided among children ages 8-12 and 13-18 and parents of children ages 2-4, 5-7, 8-12, and 13-18. Children in the 8-12 and 13-18 year age groups described and discussed their symptoms related to EoE. Three focus interviews in the 5-7 year age group were also attempted. However, children in this younger age group were not able to fully describe and discuss their symptoms. Therefore, only parent proxy PRO measures were developed for children in the 2-4 and 5-7 year old age groups. Children ages 8-12 and 13-18 were interviewed separately from their parents. Children in the 8-12 and 13-18 year groups focused on pain and difficulties with eating food. Regarding frequency, most children described this as how "often" a particular symptom was occurring. Regarding severity, all children described this as how "bad" a particular symptom was. We were surprised to find how clearly children could discern the feeling of nausea from vomiting or abdominal pain. Most children also accurately described heartburn and regurgitation. In addition, children frequently described dysphagia as trouble swallowing or the sensation of food getting stuck.

### Focus Interviews Parent Proxy-Report

Although similar to patient self-reported symptoms, parent proxy-reported concerns often differed regarding perceptions of symptom severity. In addition, parents focused more on amounts of food eaten than children did. Parents also described issues with frequency and severity of symptoms as separate from the overall question of how much of a problem a particular symptom was for their child.

### Expert Opinion

National EoE experts were particularly helpful regarding dysphagia and the various ways that patients and parents describe this important symptom. In addition to items asking about trouble swallowing and feelings of food getting stuck, items were added regarding needing to drink liquids to help swallow food and extended time needed to eat food compared to others. Expert opinions were added to the PEESS™ v2.0 draft to be reviewed by the cognitive interview cohort. No items were deleted based on expert opinion.

### Focus Interviews Item and Instrument Draft Development

Thirty-six focus interview transcripts were analyzed. Content and themes derived by consensus among the research team are summarized in Table 4. Once developed, the instrument construct, instructions, timeframe, scoring, and items were systematically reviewed with a different group of patients and their parents during the cognitive interviews.

**Table 4:** Revised items and reasons for revision

Item	Focus interviews patient reported symptoms	PEESS™ v2.0	Cognitive interviews: respondent debriefing	PEESS™ v2.0 content validated
**General**	Patients described how often symptoms occurred and how bad symptoms as different concepts All 36 subjects did not understand medical terminology in PEESS v1.0		All 39 subjects agreed that the visual analog scale for symptom severity improved comprehension	Patients preferred items in forms of questions Detailed instructions All 39 subjects preferred PEESS™ v2.0 over PEESS v1.0
**Chest Pain**	8 reported chest pain.	Chest pain, aches or hurt	All 39 participants understood the question and preferred to include the additional descriptive words.	How often do you have chest pain, ache, or hurt? How bad is the chest pain, ache, or hurt?
**Heartburn**	4 reported heartburn, 1 reported reflux, 4 reported "burning in chest"	Burning in chest, mouth, or throat (heartburn)	38 out of the 39 participants understand heartburn	How often do you have heartburn (burning in your chest, mouth, or throat)? How bad is your heartburn (burning in your chest, mouth, or throat)?
**Abdominal Pain**	5 reported abdominal pain, 8 reported "stomachaches/pain"	Stomachaches or bellyaches	All participants understood stomachaches and bellyaches. Even though it is grammatically incorrect, patients preferred the separation of stomach and belly to aches.	How often do you have stomach aches or belly aches? How bad are the stomach aches or belly aches?
**Dysphagia/** **Food Impaction**	3 reported dysphagia, 16 described "trouble swallowing" 4 reported feeling of "food getting stuck "	Trouble swallowing Needing a drink to help swallow food Needing more time to eat than other children the same age. Feeling like food gets stuck in throat or chest	All participants understood trouble swallowing. All participants understood needing a drink to help swallow food. Some participants did not know that this was a symptom of EoE. All participants understood needing more time to eat than other children the same age. Some patients did not see this as a symptom of EoE but as a behavioral issue. The participants agreed that how bad did not apply to this question, and there for only frequency was used All participants understood feeling like food gets stuck in throat or chest	How bad is the trouble swallowing? How bad is the trouble swallowing? How often do you need to drink a lot to help swallow your food? How bad is it if you don't drink a lot to help swallow your food? How often do you need more time to eat than others? Item deleted How often do you feel like food gets stuck in your throat or chest?
**Vomiting**	9 reported vomiting, 5 reported "throwing up"	Throwing up (vomiting)	All participants understood throwing up	How often do you vomit (throw up)? How bad is the vomiting (throwing up)?
**Nausea**	2 reported nausea, 3 reported "feeling like they are going to throw up but don't"	Feeling like throwing up, but didn't (nausea)	38 out of 39 participants understood the term nausea. All participants did understand the descriptive phrase for nausea.	How often do you feel nauseous (feel like you're going to throw up, but don't)? How bad is the nausea (feeling like you're going to throw up, but don't)?
**Regurgitation**	6 described as "food coming up throat".	Food coming back up throat when eating	All patients understood food coming back up throat when eating.	How often does food come back up your throat when eating? How bad is the food coming back up your throat when eating?
**Poor appetite**	13 reported poor appetite or "not wanting to eat"	Not eating as much as other children the same age	Most patients understood not eating as much as other children as a measure for poor appetite and inadequate weight gain. The participants agreed that how bad did not apply to this question, and therefore, only frequency was used	How often do you eat less than others?
**Inadequate weight gain**	1 reported inadequate weight gain		The 30 day time frame of PEESS v 2.0 does not allow for appropriate measure of inadequate weight gain.	Item Deleted
**Early Satiety**	1 reported early satiety	My child has/I have two or more of these problems this often: Other problems. They are:	Removed because only one patient reported early satiety as a symptom. All 39 subjects found this question confusing and unnecessary. It was removed in the final version. All 39 subjects found this question unnecessary as all symptoms pertaining to EoE were covered. It was removed in the final version.	Item Deleted Item Deleted Item Deleted

### Cognitive Interview Patient Self-Report

A different cohort of 39 participants divided evenly among children ages 8-12 and 13-18 and parents of children ages 2-4, 5-7, 8-12, and 13-18 were interviewed. Results from the separate cohort of children with EoE in the 8-12 and 13-18 year groups who participated in the cognitive interview respondent debriefing are summarized in Table 2. In general, participants thought that the PEESS™ version 2.0 preliminary draft was much easier to understand than the PEESS v1.0. In response to questions raised about the language of the severity score response choices, a combined pictorial scale and Likert scale was presented to participants. The feedback regarding this addition was uniformly positive, with participants asserting that the draft was much easier to interpret. For items such as vomiting, nausea, and heartburn, participants thought that having explanations of these terms in parentheses allowed for easier comprehension.

### Cognitive Interview Parent Proxy-Report

**Figure 2:**
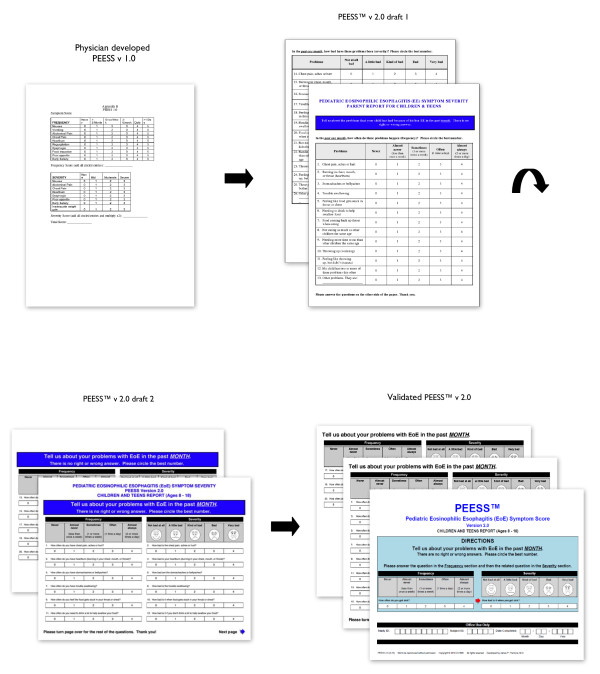
**From the PEESS v1.0 to the validated PEESS™ v 2.0**. The instrument layout and overall design went through several iterative drafts based on patient and parent-proxy feedback through focus interviews and separate cognitive interviews. For example, participants favored presenting frequency and severity of particular items side by side rather than on two separate pages.

A separate cohort of parents of children ages 2-4, 5-7, 8-12, and 13-18 was identified and interviewed independently from their children. Overall, responses to the PEESS™ version 2.0 preliminary draft were very positive, with participants reporting the response choices as much easier to understand than those in the PEESS v1.0. The addition of several questions regarding dysphagia was also positively received, and participants thought it was important to include all additional questions. The items "eating less food than others" and "needing more time to eat" were particularly important to parents of children in the 2-4 and 5-7 year age groups. Questions were raised about the overall layout of the instrument; many parents (as well as patients) thought that having frequency and severity of particular items side by side, rather than on two separate pages, would facilitate measuring each symptom comprehensively (Figure 2).

### Final Data Analysis and Construction of the PEESS™ v2.0

After the 39 cognitive interviews were concluded, each interview transcript was reviewed in conjunction with the 36 focus interview transcripts (75 total interviews). Participants' demographics are provided in Table 3. Results are illustrated in Figure 2 and summarized in Table 4. Saturation of themes and changes were deemed achieved when no new themes emerged during the focus interviews or suggestions for changes occurred during the cognitive interviews. If two or more participants did not like a particular item or shared a particular concern, the item was reviewed and modified by the research team. We modified additional language and item constructs based on Flesch Reading Easiness and Flesch-Kincaid Grade Level [17]. For example, items 5 and 6 were changed from the grammatically correct "stomachaches" and "bellyaches" to the grammatically incorrect "stomach aches" and "belly aches." This adjustment increased the Flesch Reading Ease score and decreased the Flesch-Kincaid Grade level, suggesting that the items were easier to comprehend. Disagreements were resolved by discussion, and the PEESS™ v2.0 was developed. The metrics are shown in Additional Files 1 and 2, and are available at http://www.mapi-trust.org.

## Discussion

Pediatric patient self-reported and parent proxy-reported outcomes are critical components of evaluating the impact of current and planned treatments for pediatric EoE. We report a patient self-report and parent-proxy Pediatric EoE Symptom Score (PEESS™ v2.0) metric with content-validation. The key distinction between the PEESS v1.0 compared to the PEESS™ v2.0 is that the former are physician developed metrics, while the latter was developed from the words and descriptions of patients and families following FDA guidelines published in 2009.

The variety of symptoms reported by pediatric EoE patients and their families in the development of the PEESS™ v2.0 was surprising. The existing literature reports that adult EoE patients often describe varying degrees of dysphagia, whereas children often describe pain without dysphagia as their only symptom [1,3]. Characterization of dysphagia from multiple perspectives was critical to capturing this important symptom. Patients and parent proxies were often aware that they (or their child) were experiencing dysphagia as assessed by items such as "trouble swallowing" or "food getting stuck while eating." However, the addition of items including "taking a long time to eat food" and "needing to drink a lot of water while eating food" captured the more subtle descriptions of dysphagia. Utilizing two distinct cohorts for the focus interviews and cognitive interviews also yielded invaluable information and allowed further refinement of patient self-reported and parent proxy-reported perspectives, achieving content saturation. In particular, clarification and increased reading ease of directions, addition of a combined pictorial scale and Likert scale, language modification, and changes in instrument layout were all developed from the cognitive interviews.

One particular concern regarding parent-proxy reported outcomes is that they may not be acceptable to the current FDA guidelines regarding product labeling specifications. For FDA-endorsed clinical trials that would use the PEESS™ v2.0, it may be that presently we are only able to obtain specific PRO based labeling for pediatric self-report ages 8-18 years of age. Current opinion at the FDA is that parent observer tools for children 2-7 years of age are recommended to include only symptoms clearly observable by the parent, such as: vomiting, increased time needed to eat, and drinking a large amount of liquids to swallow food. Therefore, only a subscale of questions may able to be used for clinical trials in children 2-7 years of age. However, it is also vital to recognize that FDA opinion for labeling purposes regarding PROs has changed significantly in the past several decades. It is highly possible that the current thinking on parent-proxy metrics (widely supported by many psychometricians and clinicians alike) may also change. In future studies, we will be conducting responsiveness testing of the PEESS™ v2.0 metric, further assessing the parent proxy-report metric and specific subscales. Finally, the PEESS™ v2.0 is intended for use both in and outside of the clinical trial setting. We felt that all of the symptoms important to patients and their parents as proxy are important to capture in order to reflect the true clinical picture of symptomatology.

In developing a metric for pediatric EoE, it is important to ensure that all patient areas of concern are fully elicited - a concept often termed item saturation, and a key reason why we performed our 75 patient and parent proxy interviews in two stages and two separate cohorts. Item saturation was achieved for each question and questionnaire as a whole during the cognitive interviews for each age range. Specific questions are provided in Table 2, including: "how would you change the questions words to make it easier to understand" and the overall assessment question "are there things that we forgot to ask about that you feel are important?" These questions were asked to the patients in the 8-18 year old age ranges and to the parents in the 2-18 year old age ranges. In response, the landscape questionnaire format and front page directions were changed early on, and the final draft was reviewed by patients in this format. This is an important distinction from the Flood et al. metric and the PEESS v1.0 in that items were generated by the patients in one cohort and then reviewed by a second cohort. However, the item content if the PEESS™ v2.0 is supported by the symptom overlap reported by Flood et al. and other investigators [3,15,18].

Another controversial area in PRO development is recall period. Some may argue that a one month recall period for the PEESS™ v2.0 is too long to accurately assess change. The use of a one month recall period has been well described by Varni and colleagues [19-26]. In addition, the Mayo Dysphagia Questionnaire developed by Romero and colleagues was shown not to be responsive over a 14 day recall period, but responsive over a 30 day (1 month) recall period [27]. In future studies we will be conducting responsiveness testing of the PEESS™ v2.0 metric and will be assessing different recall periods - 1 week, 2 week and 1 month. One alternative to improve patient accuracy in symptom reporting by a shorter recall period and also allow for a total responsive period of 30 days would be to collect symptom scores on a weekly basis for 4 weeks, and then report a 30 day summation score. Some have also advocated for the use of daily assessments through electronic diaries. We considered using daily electronic diaries, but we have found the patient response-burden too high, and that these diaries are impractical for any but the most highly funded and closely regulated clinical trials. The PEESS™ v2.0 is intended for use both in and outside the clinical trial setting as a simple, yet effective patient-derived metric that does not cause undue burden on patients, families, clinicians, or researchers.

There are several important limitations to this study. Although the PEESS™ v2.0 metric represents an important step forward for the field of pediatric EoE, further testing and modifications will be needed. Despite item saturation, there may be additional EoE symptoms that have not been included. In addition, generalizability to non-Caucasians and to patients with co-morbidities may be a potential concern. For the PEESS™ v2.0 metric development and cognitive interviews, we felt it critical to assess patients' and families' concerns specific to EoE, not those related to other co-morbidities. In the next phase of the PEESS™ v2.0 metric development, we will test the metric construct and reliability in over 200 patients and 250 parents across a variety of demographics and co-morbidities. The fourth phase of psychometric testing will test the responsiveness of the PEESS™ v2.0 metric, key to assessing the potential performance of the PEESS™ v2.0 in clinical trials. The PEESS™ v2.0 metrics are available at http://www.mapi-trust.org.

## Conclusions

Currently, pediatric EoE is assessed using number of eosinophils per high powered field in an esophageal endoscopic biopsy specimen as the primary outcome variable. Symptoms are at best reported as secondary outcomes utilizing physician directed questions, without any attention to PROs [2,28]. It is currently well-described that the severity of histologic inflammation in EoE as measured by tissue eosinophil counts may not directly relate to the degree of symptom severity [5]. For example, initial data in the pediatric Reslizumab (Cinquil^®^) study suggests that patient symptoms and health related quality of life (HRQOL) do not correlate with histologic esophageal inflammation [29]. In a condition without known risk of malignancy or reduced life expectancy, PROs need to be equally if not more important outcome measures compared to histologic disease activity. Face and content validation of the PEESS™ v2.0 are important first steps to establishing patient self-report and parent proxy-report symptom assessments as key factors in pediatric EoE for patients, families, researchers, and care providers alike.

## Competing interests

Cincinnati Children's Hospital Medical Center and Dr. James P. Franciosi hold the copyright and the trademark for the PEESS™ v2.0 and may receive financial compensation from the Mapi Research Trust, a nonprofit research institute that charges distribution fees to for-profit companies that use the PEESS™ v2.0™.

## Authors' contributions

JPF substantially contributed to study conception and design, as well as acquisition, analysis, and interpretation of data, drafting and revision of the manuscript, and acquisition of funding. KAH substantially contributed to study conception and design, analysis and interpretation of data, and revision of the manuscript. CWD substantially contributed to acquisition, analysis, and interpretation of data, as well as revision of the manuscript. ABG substantially contributed to study conception and design, acquisition, analysis, and interpretation of data, as well as drafting and revision of the manuscript. AJG substantially contributed to study conception and design, acquisition, analysis, and interpretation of data, as well as drafting and revision of the manuscript. JPA substantially contributed to study conception and design, revision of the manuscript, and acquisition of funding. MER contributed to analysis and interpretation of data, revision of the manuscript, and acquisition of funding. JWV substantially contributed to study conception and design and revision of the manuscript. All authors read and approved the final manuscript.

## Pre-publication history

The pre-publication history for this paper can be accessed here:

http://www.biomedcentral.com/1471-230X/11/126/prepub

## Supplementary Material

Additional File 1 **Supplement 1 - Pediatric Eosinophilic Esophagitis (EoE) Symptom Score (PEESS™ v2.0) Children and Teens Report (Ages 8-18)**. Click here for file

Additional File 2 **Supplement 2 - Pediatric Eosinophilic Esophagitis (EoE) Symptom Score (PEESS™ v2.0) Parent Report for Children and Teens (Ages 2-18)**. Click here for file
