# Supplementary material for: Development of a validated patient-reported symptom metric for pediatric Eosinophilic Esophagitis: qualitative methods
Source: BMC Gastroenterol. 2011 Nov 18;11:126. doi: 10.1186/1471-230X-11-126 (PMC3228698; doi:10.1186/1471-230X-11-126)
Supplement: Additional File 1 — Supplement 1 - Pediatric Eosinophilic Esophagitis (EoE) Symptom Score (PEESS™ v2.0) Children and Teens Report (Ages 8-18). [file 1471-230X-11-126-S1.PDF]

COPYRIGHT PROTECTED DO NOT REPRODUCE WITHOUT PERMISSION

PEESS™

Pediatric Eosinophilic Esophagitis (EoE) Symptom Score

Version 2.0

CHILDREN AND TEENS REPORT (Ages 8 - 18)

DIRECTIONS

Tell us about your problems with EoE in the past ***MONTH***.

There are no right or wrong answers. Please circle the best number.

Please answer the question in the Frequency section and then the related question in the Severity section.

Frequency				
**Never**	**Almost never** (less than once a week)	**Sometimes** (1 or more times a week)	**Often** (1 time a day)	**Almost always** (2 or more times a day)

How often do you get sick?

0	1	2	3	4

Severity				
**Not bad at all** 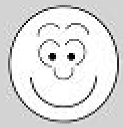	**A little bad** 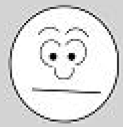	**Kind of bad** 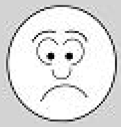	**Bad** 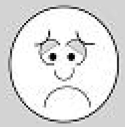	**Very bad** 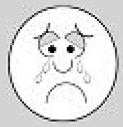

How bad is it when you get sick?

0	1	2	3	4

Office Use Only

Study ID: ☐☐☐☐☐☐☐☐☐☐ **Subject ID:** ☐☐☐☐☐ **Date Completed:** ☐☐ / ☐☐ / ☐☐☐☐

Month Day Year

 Not to be reproduced without permission Copyright © 2010 CCHMC All rights reserved Developed by James P. Franciosi, M.D.

Tell us about your problems with EoE in the past ***MONTH***.

Frequency				
**Never**	**Almost never** (less than once a week)	**Sometimes** (1 or more times a week)	**Often** (1 time a day)	**Almost always** (2 or more times a day)

1. How often do you have chest pain, ache, or hurt?

0	1	2	3	4

3. How often do you have heartburn (burning in your chest, mouth, or throat)?

0	1	2	3	4

5. How often do you have stomach aches or belly aches?

0	1	2	3	4

7. How often do you have trouble swallowing?

0	1	2	3	4

9. How often do you feel like food gets stuck in your throat or chest?

0	1	2	3	4

11. How often do you need to drink a lot to help swallow your food?

0	1	2	3	4

13. How often do you vomit (throw up)?

0	1	2	3	4

15. How often do you feel nauseous (feel like you're going to throw up, but don't)?

0	1	2	3	4

Severity				
**Not bad at all** 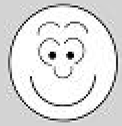	**A little bad** 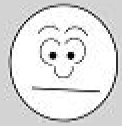	**Kind of bad** 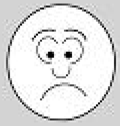	**Bad** 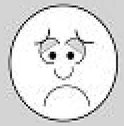	**Very bad** 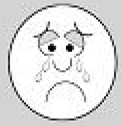

2. How bad is the chest pain, ache, or hurt?

0	1	2	3	4

4. How bad is your heartburn (burning in your chest, mouth, or throat)?

0	1	2	3	4

6. How bad are the stomach aches or belly aches?

0	1	2	3	4

8. How bad is the trouble swallowing?

0	1	2	3	4

10. How bad is it when food gets stuck in your throat or chest?

0	1	2	3	4

12. How bad is it if you don't drink a lot to help swallow your food?

0	1	2	3	4

14. How bad is the vomiting (throwing up)?

0	1	2	3	4

16. How bad is the nausea (feeling like you're going to throw up, but don't)?

0	1	2	3	4

COPYRIGHT PROTECTED
DO NOT REPRODUCE
WITHOUT PERMISSION

Please turn to the next page for the rest of the questions. Thank you!

Next page

PEESS 2.0 (8-18) Not to be reproduced without permission Copyright © 2010 CCHMC All rights reserved Developed by James P. Franciosi, M.D.

Tell us about your problems with EoE in the past ***<u>MONTH</u>***.

Frequency

**Never**	**Almost never**<br><br>(less than once a week)	**Sometimes**<br><br>(1 or more times a week)	**Often**<br><br>(1 time a day)	**Almost always**<br><br>(2 or more times a day)

Severity

**Not bad at all**	**A little bad**	**Kind of bad**	**Bad**	**Very bad**
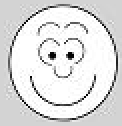	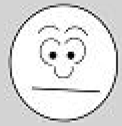	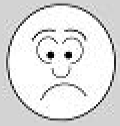	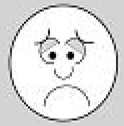	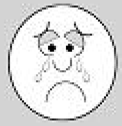

17. How often does food come back up your throat when eating?

0	1	2	3	4

18. How bad is the food coming back up your throat when eating?

0	1	2	3	4

19. How often do you eat less food than others?

0	1	2	3	4

20. How often do you need more time to eat than others?

0	1	2	3	4

Thank you very much for participating!

COPYRIGHT PROTECTED DO NOT REPRODUCE WITHOUT PERMISSION

PEESS 2.0 (8-18) Not to be reproduced without permission Copyright © 2010 CCHMC All rights reserved Developed by James P. Franciosi, M.D.
