# Supplementary material for: Development of a validated patient-reported symptom metric for pediatric Eosinophilic Esophagitis: qualitative methods
Source: BMC Gastroenterol. 2011 Nov 18;11:126. doi: 10.1186/1471-230X-11-126 (PMC3228698; doi:10.1186/1471-230X-11-126)
Supplement: Additional File 2 — Supplement 2 - Pediatric Eosinophilic Esophagitis (EoE) Symptom Score (PEESS™ v2.0) Parent Report for Children and Teens (Ages 2-18). [file 1471-230X-11-126-S2.PDF]

COPYRIGHT PROTECTED
DO NOT REPRODUCE
WITHOUT PERMISSION

PEESS™

Pediatric Eosinophilic Esophagitis (EoE) Symptom Score

Version 2.0

PARENT REPORT FOR CHILDREN AND TEENS (Ages 2 - 18)

DIRECTIONS

Tell us about your child's problems with EoE in the past *MONTH*.

There are no right or wrong answers. Please circle the best number.

Please answer the question in the Frequency section and then the related question in the Severity section.

Frequency				
**Never**	**Almost never** (less than once a week)	**Sometimes** (1 or more times a week)	**Often** (1 time a day)	**Almost always** (2 or more times a day)
**How often does your child get sick?**				
0	1	2	3	4

Severity				
**Not bad at all** 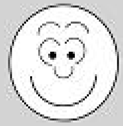	**A little bad** 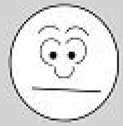	**Kind of bad** 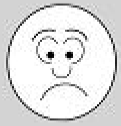	**Bad** 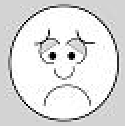	**Very bad** 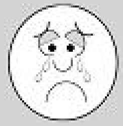
**How bad is it when your child gets sick?**				
0	1	2	3	4

Office Use Only

Study ID: ☐☐☐☐☐☐☐☐☐ **Subject ID:** ☐☐☐☐☐ **Date Completed:** ☐☐ / ☐☐ / ☐☐☐☐

Month Day Year

Not to be reproduced without permission Copyright © 2010 CCHMC All rights reserved Developed by James P. Franciosi, M.D.

Tell us about your child's problems with EoE in the past ***<u>MONTH</u>***.

Frequency				
**Never**	**Almost never** (less than once a week)	**Sometimes** (1 or more times a week)	**Often** (1 time a day)	**Almost always** (2 or more times a day)

Severity				
**Not bad at all** 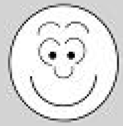	**A little bad** 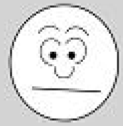	**Kind of bad** 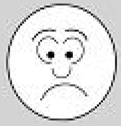	**Bad** 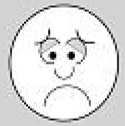	**Very bad** 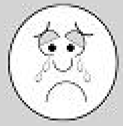

1. How often does your child have chest pain, ache, or hurt?

0	1	2	3	4

2. How bad is your child's chest pain, ache, or hurt?

0	1	2	3	4

3. How often does your child have heartburn (burning in the chest, mouth, or throat)?

0	1	2	3	4

4. How bad is your child's heartburn (burning in the chest, mouth, or throat)?

0	1	2	3	4

5. How often does your child have stomach aches or belly aches?

0	1	2	3	4

6. How bad are your child's stomach aches or belly aches?

0	1	2	3	4

7. How often does your child have trouble swallowing?

0	1	2	3	4

8. How bad is your child's trouble swallowing?

0	1	2	3	4

9. How often does your child feel like food gets stuck in his/her throat or chest?

0	1	2	3	4

10. How bad is it when your child gets food stuck in his/her throat or chest?

0	1	2	3	4

11. How often does your child need to drink a lot to help swallow food?

0	1	2	3	4

12. How bad is it when your child needs to drink a lot to help swallow food?

0	1	2	3	4

13. How often does your child vomit (throw up)?

0	1	2	3	4

14. How bad is your child's vomiting (throwing up)?

0	1	2	3	4

15. How often does your child feel nauseous (feel like throwing up, but doesn't)?

0	1	2	3	4

16. How bad is your child's nausea (feeling like throwing up, but doesn't)?

0	1	2	3	4

COPYRIGHT PROTECTED
DO NOT REPRODUCE
WITHOUT PERMISSION

Please turn to the next page for the rest of the questions. Thank you!

Next page

 Not to be reproduced without permission Copyright © 2010 CCHMC All rights reserved Developed by James P. Franciosi, M.D.

Tell us about your child's problems with EoE in the past ***<u>MONTH</u>***.

Frequency				
**Never**	**Almost never** (less than once a week)	**Sometimes** (1 or more times a week)	**Often** (1 time a day)	**Almost always** (2 or more times a day)

Severity				
**Not bad at all** 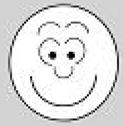	**A little bad** 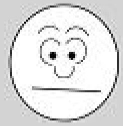	**Kind of bad** 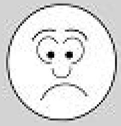	**Bad** 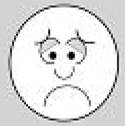	**Very bad** 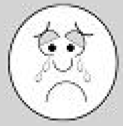

17. How often does your child have food come back up in his/her throat when eating?

0	1	2	3	4

18. How bad is it when food comes back up in your child's throat?

0	1	2	3	4

19. How often does your child eat less than others?

0	1	2	3	4

20. How often does your child need more time to eat than others?

0	1	2	3	4

Thank you very much for participating!

COPYRIGHT PROTECTED
DO NOT REPRODUCE WITHOUT PERMISSION

 Not to be reproduced without permission Copyright © 2010 CCHMC All rights reserved Developed by James P. Franciosi, M.D.
